# Association between Changes in White Matter Microstructure and Cognitive Impairment in White Matter Lesions

**DOI:** 10.3390/brainsci12040482

**Published:** 2022-04-07

**Authors:** An-Ming Hu, Yan-Ling Ma, Yue-Xiu Li, Zai-Zhu Han, Nan Yan, Yu-Mei Zhang

**Affiliations:** 1 Department of Rehabilitation Medicine, Beijing Tiantan Hospital, Capital Medical University, Beijing 100070, China; huanming@mail.ccmu.edu.cn (A.-M.H.); liyuexiu@mail.ccmu.edu.cn (Y.-X.L.); 2 Beijing Xiaotangshan Hospital, Beijing 102211, China; mayl2009@163.com; 3 State Key Laboratory of Cognitive Neuroscience and Learning & IDG/McGovern Institute for Brain Research, Beijing Normal University, Beijing 100875, China; zzhhan@bnu.edu.cn; 4 CAS Key Laboratory of Human-Machine Intelligence-Synergy Systems, Shenzhen Institute of Advanced Technology, Chinese Academy of Sciences, Shenzhen 518055, China

**Keywords:** white matter lesions, cognitive impairment, tract-based spatial statistics, white matter microstructure

## Abstract

This study investigated the characteristics of cognitive impairment in patients with white matter lesions (WMLs) caused by cerebral small vessel disease and the corresponding changes in WM microstructures. Diffusion tensor imaging (DTI) data of 50 patients with WMLs and 37 healthy controls were collected. Patients were divided into vascular cognitive impairment non-dementia and vascular dementia groups. Tract-based spatial statistics showed that patients with WMLs had significantly lower fractional anisotropy (FA) and higher mean diffusivity (MD), axial diffusivity (AD), and radial diffusivity (RD) values throughout the WM areas but predominately in the forceps minor, forceps major (FMA), bilateral corticospinal tract, inferior fronto-occipital fasciculus, superior longitudinal fasciculus, inferior longitudinal fasciculus (ILF), and anterior thalamic radiation, compared to the control group. These fiber bundles were selected as regions of interest. There were significant differences in the FA, MD, AD, and RD values (*p* < 0.05) between groups. The DTI metrics of all fiber bundles significantly correlated with the Montreal Cognitive Assessment (*p* < 0.05), with the exception of the AD values of the FMA and ILF. Patients with WMLs showed changes in diffusion parameters in the main WM fiber bundles. Quantifiable changes in WM microstructure are the main pathological basis of cognitive impairment, and may serve as a biomarker of WMLs.

## 1. Introduction

White matter lesions (WMLs), also known as leukoaraiosis, are characterized by low-density periventricular shadows on computed tomography, and high signals on magnetic resonance imaging (MRI) T2-weighted images and fluid-attenuated inversion recovery (FLAIR) sequences [1]. WMLs are a common cause of vascular cognitive impairment, which often manifest as an impairment in executive function, delayed recall, attention deficits, and a decrease in information processing speed. In the early stage, patients exhibit cognitive impairment (i.e., vascular cognitive impairment non-dementia) with gradual progression of the disease. In severe cases, dementia is evident [2,3]. WM changes are crucial pathological features in patients with WMLs; thus, recognizing the relationship between changes in WM and cognitive impairment will provide important opportunities to prevent brain damage [4].

Many studies have used diffusion tensor imaging (DTI) to investigate the microstructure and integrity of WM and the relationship between changes in WM and cognitive impairment in WMLs. Previous studies have shown that the characteristic pattern of DTI in WMLs is consistent with axonal loss and gliosis leading to impairment and loss of directional diffusion [5]. DTI can reflect the microstructure integrity, damage to WM, and contribution to accumulating brain damage in the form of diffusion parameters, by measuring the diffusion of water molecules in the tissues [6]. The parameters mainly include fractional anisotropy (FA), mean diffusivity (MD), axial diffusivity (AD), and radial diffusivity (RD) [7,8]. FA is generally used to reflect the integrity of fiber structure, which is related to the integrity of the fiber axon and myelin sheath and the density and running of the fiber bundle. MD measures the bulk mobility of water molecules, which reflects the diffusion level and diffusion resistance of the whole molecule [9]. AD reflects the diffusivity of water molecules along the long axis of the fiber bundle, whereas RD reflects the diffusivity of water molecules in the direction perpendicular to the long axis of the fiber bundle [10,11].

Some studies have also confirmed that cognitive function is closely related to the integrity of WM detected by DTI [12,13,14]. The disruption of normal-appearing WM (NAWM) integrity reflected by DTI parameters correlates more strongly with psychomotor dysfunction than WM hyperintensity load because NAWM occupies the main body of global WM [15]. The integrity of the structure and function of WM fibers is critical for information exchange and cooperation among brain regions.

Quantifiable DTI parameters are sensitive indicators for evaluations of disease progression and cognitive impairment [16,17,18]. Quantitative analysis methods using DTI data include a region of interest (ROI) analysis, voxel-based analysis (VBA), and tract-based spatial statistics (TBSS) [19]. Based on voxels, the dispersion parameters of each subject are projected onto the WM skeleton for comparison with TBSS analysis. This method overcomes the subjectivity and non-repeatability of ROI and the lack of a unified standard for the Gaussian kernel smoothing size of VBA.

To the best of our knowledge, few TBSS studies have been performed to investigate diffusion measurement differences between WMLs patients with vascular cognitive impairment non-dementia (VCIND) and vascular dementia (VaD). In this study, we investigated the characteristics of cognitive impairment in patients with WMLs caused by cerebral small vessel disease (CSVD) and the corresponding changes in WM microstructures. A combination method of TBSS and ROI was used to determine changes in WM microstructure related to different degrees of cognitive impairment. The clinical significance of the DTI findings through correlations with cognitive evaluations was also explored.

## 2. Materials and Methods

### 2.1. Ethical Approval

This study was in accordance with the policies set by the Declaration of Helsinki, and the research procedure was approved by the Ethic Committees of Beijing Tiantan Hospital, Capital Medical University, Beijing China (Approval No. KYSB2016.023). Formal written informed consent was obtained from all participants.

### 2.2. Participants

Outpatients who underwent MRI at Beijing Tiantan Hospital between 2014 and 2018 were retrospectively recruited. Some patients went to the hospital with symptoms of cognitive decline or dizziness, whereas others went for physical examination. Two radiologists independently and unanimously diagnosed recruited patients with WMLs caused by CSVD according to the Standards for Reporting Vascular Changes on Euroimaging (STRIVE) criteria [20]. They were blinded to the clinical profiles of patients, and visually evaluated the FLAIR magnetic resonance images without any information about the participants.

According to a revised version of the Fazekas scale, the inclusion criteria for patients with WMLs were the presence of WMLs on MRI scans and age between 40 and 85 years. The healthy controls (HCs) aged between 40 and 85 were recruited from neighboring communities. The exclusion criteria for both WMLs patients and HCs were: patients with cardiac or renal failure, cancer, or other severe systemic diseases; patients with unrelated neurological diseases such as epilepsy, traumatic brain injury, and multiple sclerosis; patients with chronic cerebral infarction or other lesions; patients with leukoencephalopathy or dementia of non-vascular origin; patients with psychiatric diseases or drug addiction; patients with consciousness disruption or aphasia; and the inability or refusal to undergo brain MRI.

### 2.3. Clinical Data Collection

Basic information on all participants was collected, including age, sex, education years, hypertension, diabetes mellitus, lipoprotein metabolism disorders, and other basic clinical information.

### 2.4. Cognitive Measures

Cognitive functions were evaluated by a trained neurologist in a quiet room without external interference. The Montreal Cognitive Assessment (MoCA) was used to screen overall cognitive function [21]. The MoCA evaluates seven cognitive domains: (1) visuospatial, which is assessed by a clock-drawing task, a three-dimensional cube copy, and an alternation task adapted from the Trail Making B task; (2) naming, which is assessed by a three-item confrontation naming task; (3) attention, which is assessed using target detection using tapping and forward and backward digit span; (4) language, which is assessed by a phonemic fluency task and using the repetition of two syntactically complex sentences; (5) abstraction, which is assessed using a verbal abstraction task; (6) memory, which involves two learning trials of five nouns and delayed recall; and (7) orientation, in which place and time are evaluated [22]. For patients with cognitive impairment, the Clinical Dementia Rating (CDR) score was used to assess impairment severity [23]. According to the diagnostic criteria of the National Institute of Neurological Disorders and Stroke/Swiss Society for Neuroscience, for vascular dementia, patients were divided into the VCIND and VaD groups [23].

WML-VCIND group: WMLs were consistently observed on MRI (CDR = 0.5 and MoCA < 26).

WML-VaD group: WMLs were consistently observed on MRI, (CDR ≥ 1 and MoCA < 26).

### 2.5. Image Acquisition

The MRI data were acquired on a 3.0-T Siemens scanner. Participants lay in a supine position with their heads snugly fixed by a belt and foam pads to minimize head motion. DTI images were acquired using a single-shot, twice-refocused, diffusion-weighted echo planar imaging sequence with the following scan parameters: repetition time (TR) = 8000 ms; echo time (TE) = 96 ms; 64 diffusion-weighted directions with a b value of 1000 s/mm^2^ and 11 images with a b value of 0 s/mm^2^; flip angle = 90°; field of view = 224 mm^2^; in-plane resolution = 1.75 mm × 1.75 mm voxels; and 54 contiguous 2 mm thick axial slices.

### 2.6. DTI Data Pre-Processing

DTI image pre-processing was performed using PANDA software (a pipeline tool for analyzing brain diffusion images; http://www.nitrc.org/projects/panda/, accessed on 3 March 2021). After briefly converting DICOM files to NIFTI images, estimating the brain mask, cropping the raw images to reduce image size and memory cost, and correcting for the eddy-current effect and head motion artifacts, main diffusion metrics (i.e., FA, MD, AD and RD) were successfully calculated [24].

### 2.7. TBSS Analysis

Subsequently, TBSS analysis was conducted. This method was used to detect WM microstructure, which significantly differed between patients and healthy controls (HCs). The regions with significant differences were considered ROIs.

The following five steps were initially performed on the FA images. Detailed processes were presented in the paper by Smith et al. [19]. (1) The FA image of each subject was aligned to a preidentified target FA image (FMRIB58_FA) using non-linear image registration algorithm. (2) All aligned FA images were transformed onto the MNI152 template using affine registration. (3) Mean FA skeleton was produced from the images of all the subjects. (4) Individual subject FA images were presented to the skeleton. (5) Voxel-wise statistics, across subjects, were calculated for each point on the common skeleton. Subsequently, data on MD, AD, and RD were created similarly by applying the same steps outlined above. The statistical analyses of these diffusion tensor metrics were performed, similar to the FA analyses.

### 2.8. Statistical Analyses

Statistical Package for the Social Sciences (version 19.0; IBM Corp., Armonk, NY, USA) was used for data processing. The measurement variables in the general data follow a normal distribution and are reported as ($\bar{x}$ ± s). A one-way analysis of variance (ANOVA) was used for intergroup comparisons. Subsequently, post hoc Least Significant Difference tests were performed to compare the differences among the three groups. The count data are expressed as frequencies and were compared between groups using the χ^2^ test. *p* < 0.05 was considered statistically significant.

### 2.9. TBSS Voxel-Wise Statistical Analyses

The F-test (intergroup differences, no repeated measures) design based on the general linear model in the FMRIB software library (https://fsl.fmrib.ox.ac.uk/fsl/fslwiki, accessed on 5 March 2021) combined with a permutation-based inference tool for non-parametric statistical thresholding (the “randomise” tool) was used to compare differences among the WML-VCIND, WML-VaD, and HC groups. *p* < 0.05 was considered statistically significant (family-wise error corrected for multiple comparisons) using the threshold-free cluster enhancement (TFCE) option in the “randomise” permutation-testing tool.

### 2.10. ROI-Wise Statistical Analysis

The “JHU White-Matter Tractography Atlas” in the standard space allows for the parcellation of the entire FA skeleton into multiple ROIs, from which our focus white matter tracts were selected. The ROIs were selected based on the TBSS results and analysis of covariance (ANCOVA) was performed to compare the resultant ROI-based DTI metrics among the WML-VCIND, WML-VaD, and HC groups, controlling the impact of age, sex, and education years. Bonferroni correction was applied to correct for multiple comparisons. Subsequently, the post hoc test was performed to compare group differences among groups. Lastly, a partial correlation analysis was used to calculate the correlation between the resultant between-group different ROI-based data and MoCA performance, with age, sex, and education years as covariates. *p* < 0.05 was considered statistically significant.

## 3. Results

### 3.1. Participant Characteristics

There were significant differences in the MoCA scores between patients with WMLS and healthy controls (HC) (*p* < 0.001). Post hoc results showed that the MoCA score was higher in patients with patients with WML-VCIND than in patients with WML-VaD (*p* < 0.05). There was no significant difference in sex and education years between the WMLS and control groups. There was no significant difference in age between the WML-VCIND and WML-VaD groups (*p* > 0.05; Table 1). It is worth noting that the effects of age, sex, and education were eliminated in subsequent cognitive and WM microstructure integrity analyses.

### 3.2. TBSS Analysis of DTI Data

**Figure 1:**
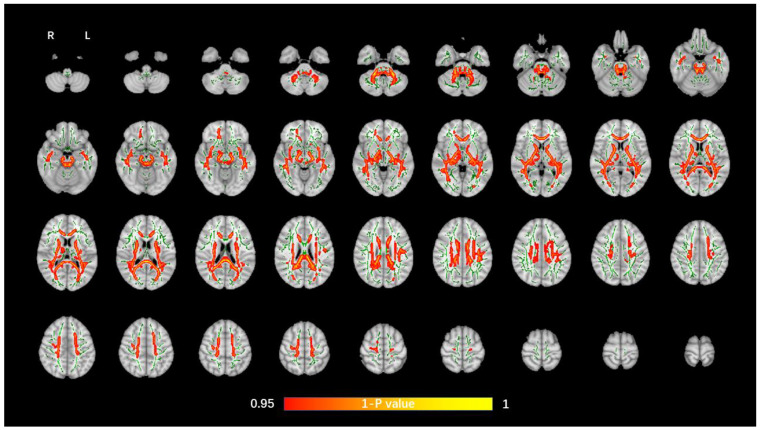
Voxel-wise TBSS analysis results of FA images among the WML-VCIND, WML-VaD, and HC groups. Green represents the mean WM skeleton of all subjects. Red-yellow (thickened for better visibility) represents regions with a significant F-test statistical difference (*p* < 0.05, TFCE-based FWE-corrected). TBSS, tract-based spatial statistics; FA, fractional anisotropy; HC, healthy controls; WML, white matter lesions; WML-VCIND, WML and non-dementia vascular cognitive impairment; WML-VaD, WML and vascular dementia.

**Figure 2:**
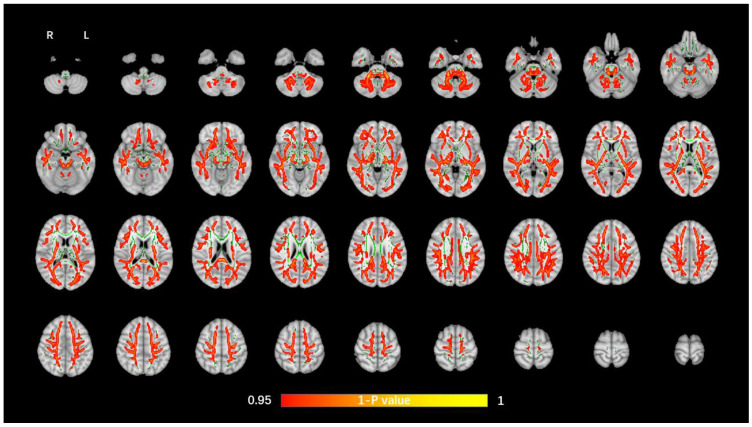
Voxel-wise TBSS analysis results of MD images among the WML-VCIND, WML-VaD, and HC groups. Green represents the mean WM skeleton of all subjects. Red-yellow (thickened for better visibility) represents regions with a significant F-test statistical difference (*p* < 0.05, TFCE-based FWE-corrected). TBSS, tract-based spatial statistics; MD, mean diffusivity.

**Figure 3:**
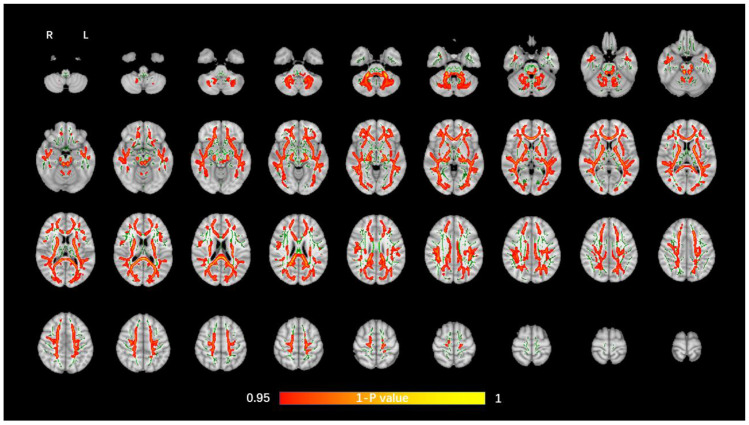
Voxel-wise TBSS analysis results of AD images among the WML-VCIND, WML-VaD, and HC groups. Green represents the mean WM skeleton of all subjects. Red-yellow (thickened for better visibility) represents regions with a significant F-test statistical difference (*p* < 0.05, TFCE-based FWE-corrected). TBSS, tract-based spatial statistics; AD, axial diffusivity.

**Figure 4:**
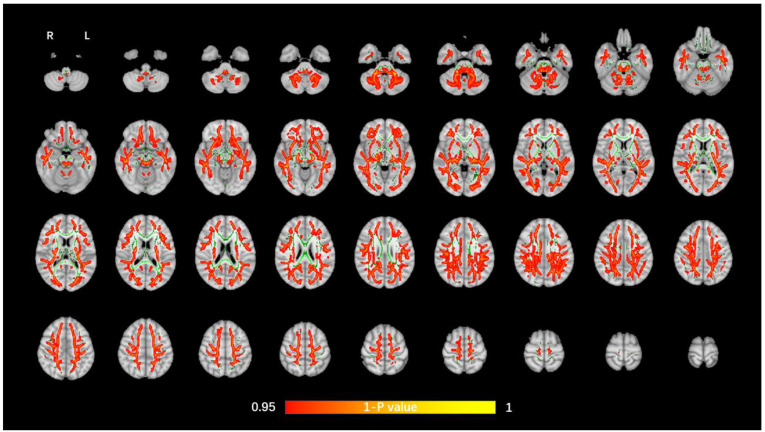
Voxel-wise TBSS analysis results of RD images among the WML-VCIND, WML-VaD, and HC groups. Green represents the mean WM skeleton of all subjects. Red-yellow (thickened for better visibility) represents regions with a significant F-test statistical difference (*p* < 0.05, TFCE-based FWE-corrected). TBSS, tract-based spatial statistics; RD, radial diffusivity.

The average FA WM skeleton of all subjects was constructed in TBSS analyses, as shown by the green line in Figure 1, Figure 2, Figure 3 and Figure 4. An analysis of variance of voxel level was carried out on the WM skeleton, and the differences among the three groups were compared. *p* < 0.05 after FWE correction based on TFCE was statistically significant. Statistically significant areas were expanded to better show the position of WM fiber bundles, as shown in the red part in Figure 1, Figure 2, Figure 3 and Figure 4. The statistical results showed that the DTI indexes of most fiber bundles were statistically significant. There was a significant statistical difference in the FA, MD, AD, and RD values of the FMI, FMA, IFOF, SLF, ILF, ATR and corticospinal tract (CCT) among the three groups (*p* < 0.05; Figure 1, Figure 2, Figure 3 and Figure 4).

### 3.3. ROI Analyses

Combined with the WM partition map “JHU White-Matter Tractography Atlas”, the whole brain FA WM skeleton was partitioned, and 10 fiber bundles with significant differences as observed in the TBSS voxel level analyses were selected as ROIs: the forceps minor (FMI); forceps major (FMA); IFOF.L, IFOF.R; SLF.L, SLF.R; ILF.L, ILF.R; and ATR.L, ATR.R. Although differences in CCT were found in the TBSS analysis, we did not select it as an ROI considering that CCT and motor function were clearly correlated.

**Figure 5:**
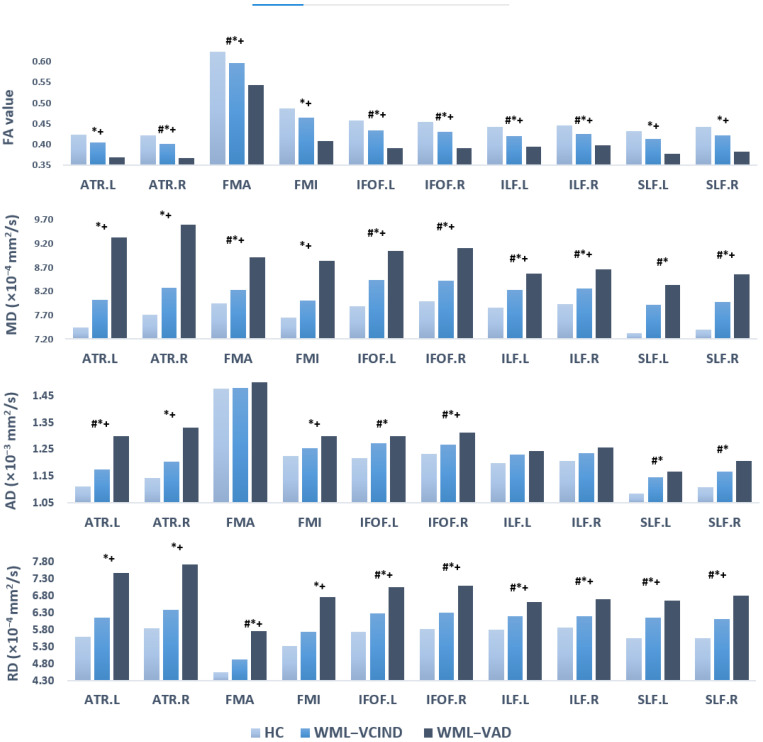
DTI index of ROI level analysis results of WM skeleton partition. # *p* < 0.05 post hoc result for WML-VCIND vs. HC. * *p* < 0.05 post hoc result for WML-VaD vs. HC. + *p* < 0.05 post hoc result for WML-VaD vs. WML-VCIND. HC, healthy controls; WML, white matter lesions; WML-VCIND, WML and non-dementia vascular cognitive impairment; WML-VaD, WML and vascular dementia.

ANCOVA analyses showed that there were significant differences in FA, MD, AD, and RD values (*p* < 0.05), which were mutually confirmed with the results of TBSS voxel level analyses. Post hoc results showed that compared to the HC group, the WML-VAD group had significantly lower FA and higher MD, AD, and RD; the WML-VCIND group also had significantly lower FA and higher MD, AD, and RD. A few other fiber bundles were not significantly different between groups. Compared to the WML-VCIND group, most fiber bundles in the WML-VAD group also showed significant differences between groups. The FA in the WML-VAD group was significantly lower, whereas the MD, AD, and RD were higher. A small number of fibers with no significant differences between groups also showed similar trends (Table 2, Table 3, Table 4 and Table 5, Figure 5).

### 3.4. Correlation Analysis Results between the DTI Index in ROI and MoCA

**Figure 6:**
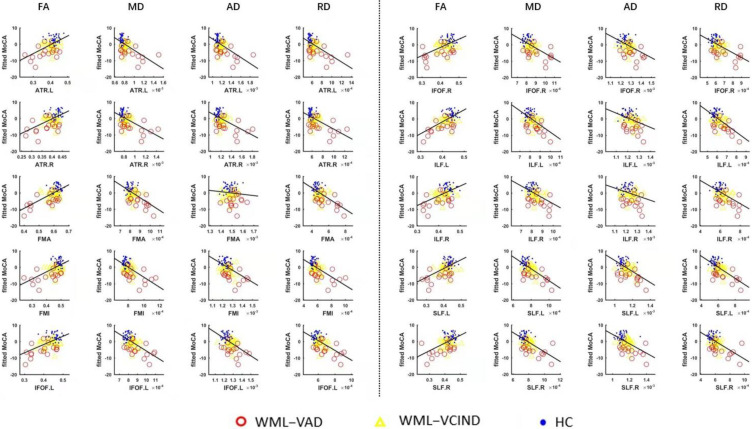
Correlation analysis results of DTI index in ROI and MoCA.

The correlation between the mean value of the DTI index and MoCA in the 10 WM fiber bundles was analyzed. The partial correlation analysis method was used to control the effects of sex, age, and education on the statistical results. The results showed that, with the exception of the AD values of FMA and ILF.R, the DTI indexes of all fiber bundles were significantly correlated with MoCA (*p* < 0.05; Table 6, Figure 6).

## 4. Discussion

CSVD is a common cause of cognitive impairment and VaD. Correlations between CSVD imaging characteristics and cognitive functions have been detected in various settings [20,25]. In this study, we showed extensive damage to the main WM fiber bundle microstructure in patients with WMLs caused by CSVD. The present study differs from previous research in a few ways. First, few TBSS studies have been performed to investigate diffusion measurement differences in WML patients. Furthermore, previous studies have mainly focused on patients with VCIND WMLs or with mixed degrees of cognitive impairment [26,27]. We recruited homogeneous groups of patients with VCIND or VaD. Finally, the results of the VCIND group confirmed previous findings [28]. Significant differences in new diffusion parameters were also found between the WML VCIND and VaD groups.

DTI is a useful and unique tool with which to discover changes in brain WM microstructure. The TBSS method was applied to DTI data to identify WM abnormalities in the center of major WM tracts. Recent studies have used the TBSS method to evaluate microstructural changes in major WM tracts in patients with VCIND [28]. They showed that the VCIND group had decreased FA and increased MD values throughout widespread WM areas, predominately in the ATR, FMI, IFOF, ILF, and SLF. Our study also confirmed extensive changes in WM microstructure of patients with VCIND. There were slight inconsistencies in that we found significant changes in the FMA but no significant changes in the FMI between patients with VCIND and HCs. Moreover, our study found that the VaD group had decreased FA and increased MD/AD/RD values in the FMI.

The abnormalities of major WM tract can be more accurately identified with TBSS analysis. Compared to the HC group, major WM tract abnormalities in the VCIND group were predominately in the ATR, ILF, IFOF, SLF, and FMA. ATR is emitted from the anterior limb of the internal capsule, from which many fibers travel to the prefrontal lobe and cingulate gyrus, which are important components of the subcortical circuit of the frontal lobe [24]. An ATR lesion can lead to impairment of the prefrontal striatal circuit, resulting in the impairment of cognitive function [29]. ILF connects directly to the anterior temporal lobe and occipital lobe, and indirectly to the frontal lobe by connecting to the uncinate fasciculus. ILF is mainly involved in visual perception, object recognition, and other vision-related functions. It connects the ventral attention network composed of the frontal-parietal lobe and participates in goal-oriented behavior and interference elimination [30]. IFOF partially overlaps ILF and directly connects the frontal and occipital lobes from the outside through the middle of the temporal lobe. The SLF connects the frontal, parietal, occipital, and temporal lobes. The complex structure of SLF determines the complexity of its function and involvement in spatial attention, eye movement function, somatosensory information transmission between the parietal lobe and motor cortex, language pronunciation, auditory information integration, and other functions [31,32,33].

The corpus callosum (CC) is responsible for connecting the left and right cerebral hemispheres. Changes in different regions of the CC have been detected and progression of CC loss is also more rapid in individuals with WMLs who develop dementia [30]. FMA originates from the splenium of the corpus callosum, which plays an essential role in the process of transmitting and integrating the visual information of words, objects, and faces in the left/right visual field. FMI is a connecting pathway of the bilateral frontal lobes through the genu of CC. It is considered to control executive function as well as hemispheric specialization and interactions [34]. Although CC is particularly prone to damage in VCIND and VaD with WMLs, the relationship between the fiber damage of CC and the severity of cognitive impairment remains controversial [35]. Our study using a combination method of TBSS and ROI suggests that FMA may change in the early stage of cognitive impairment while FMI changes obviously when cognitive impairment develops into dementia.

This study also found corticospinal tract-related changes, which originate from the primary cortical motor neurons of the corticospinal tract and mainly involve autonomic movement. Patients with WMLs always have movement disorders, mainly involving gait and balance, along with cognitive impairment such as executive function and attention [36].

Many studies have shown that DTI changes are correlated with cognitive scale scores, and that changes in WM microstructure can affect cognitive function [37,38,39]. Our results also confirmed that both cognitive impairment and dementia were associated with abnormal WM microstructure, which may explain the poor social and behavioral performance of patients with WMLs.

This study had several limitations. First, it was cross-sectional, and longitudinal studies are needed to assess the dynamic changes in WM microstructure and cognitive impairment. Second, the sample size in each group was relatively small; thus, a larger sample size is needed to study microstructure changes in WMLs. Third, this study explored the correlation between the cognitive function of MoCA assessment and WM microstructure changes. More comprehensive cognitive assessments are needed to clarify the mechanism underlying specific neuropsychological dysfunction, such as impaired attentional function.

## 5. Conclusions

Patients with WMLs showed changes in diffusion parameters in the main WM fiber bundles; with the aggravation of cognitive impairment, the relevant parameters changed accordingly, suggesting that quantifiable changes in WM microstructure are the main pathological basis of cognitive impairment, which may serve as a biomarker of WMLs.

## Figures and Tables

**Table 1:** Patient demographic characteristics.

	HC (*n* = 37)	WML-VCIND (*n* = 33)	WML-VaD (*n* = 17)	*p*-Value
Age (years)	58.84 ± 7.80	64.00 ± 9.40 ^#^	65.12 ± 9.10 *	0.015 ^b^
Sex (male/female, *n*)	20/17	16/17	14/3	0.062 ^a^
Education level (years)	12.68 ± 2.84	11.55 ± 2.54	11.76 ± 2.97	0.209 ^b^
Hypertension (*n*)	24	24	7	0.087 ^a^
Diabetes (*n*)	31	27	14	0.975 ^a^
Hyperlipemia (*n*)	30	26	14	0.948 ^a^
MoCA score	27.41 ± 1.41	21.84 ± 2.49 ^#^	17.71 ± 4.05 *^,†^	<0.001 ^b^
Visuospatial/Executive	4.59 ± 1.04	3.41 ± 1.17 ^#^	2.18 ± 0.59 *^,†^	<0.001 ^b^
Naming	2.97 ± 0.16	2.74 ± 0.63	2.50 ± 0.73 *	0.006 ^b^
Attention	5.89 ± 0.39	5.06 ± 1.15 ^#^	4.12 ± 1.45 *^,†^	<0.001 ^b^
languge	2.45 ± 0.60	2.03 ± 0.54 ^#^	1.43 ± 0.89 *^,†^	<0.001 ^b^
abstraction	1.81 ± 0.56	1.38 ± 0.80 ^#^	1.12 ± 0.80 *	0.002 ^b^
Delayed recall	3.70 ± 0.99	1.61 ± 1.28 ^#^	1.43 ± 1.03 *	<0.001 ^b^
Orientation	6.00 ± 0.00	5.61 ± 0.71	4.93 ± 1.23 *^,†^	<0.001 ^b^

^a^ The *p* value was obtained by χ^2^ test. ^b^ The *p* value was obtained by one-way ANOVA. ^#^ *p* < 0.05 post hoc result for WML-VCIND vs. HC. * *p* < 0.05 post hoc result for WML-VaD vs. HC. ^†^  *p* < 0.05 post hoc result for WML-VaD vs. WML-VCIND. HC, healthy controls; WML, white matter lesions; WML-VCIND, WML and non-dementia vascular cognitive impairment; WML-VaD, WML and vascular dementia; MoCA, the Montreal Cognitive Assessment.

**Table 2:** ROI level analysis FA results of WM skeleton partition.

WM Tracts		FA (Mean ± Std)			ANCOVA		Post Hoc Test (Bonferroni)		
							WML-VCIND/HC	WML-VAD/HC	WML-VAD/WML-VCIND
		HC (*n* = 37)	WML-VCIND (*n* = 33)	WML-VAD (*n* = 17)	F	*p*	*p*	*p*	*p*
1	ATR.L	0.424 ± 0.023	0.406 ± 0.029	0.369 ± 0.059	12.636	<0.001 *	0.09	<0.001 *	0.002 *
2	ATR.R	0.422 ± 0.022	0.402 ± 0.024	0.367 ± 0.054	15.464	<0.001 *	0.032 *	<0.001 *	0.001 *
3	FMA	0.625 ± 0.024	0.596 ± 0.031	0.544 ± 0.084	16.292	<0.001 *	0.025 *	<0.001 *	0.001 *
4	FMI	0.487 ± 0.021	0.466 ± 0.033	0.408 ± 0.079	16.403	<0.001 *	0.117	<0.001 *	<0.001 *
5	IFOF.L	0.459 ± 0.026	0.435 ± 0.033	0.392 ± 0.056	14.784	<0.001 *	0.021 *	<0.001 *	0.001 *
6	IFOF.R	0.454 ± 0.022	0.43 ± 0.031	0.392 ± 0.061	12.607	<0.001 *	0.019 *	<0.001 *	0.002 *
7	ILF.L	0.442 ± 0.019	0.42 ± 0.025	0.395 ± 0.047	13.024	<0.001 *	0.004 *	<0.001 *	0.014 *
8	ILF.R	0.445 ± 0.021	0.426 ± 0.03	0.398 ± 0.049	9.717	<0.001 *	0.033 *	<0.001 *	0.011 *
9	SLF.L	0.433 ± 0.021	0.413 ± 0.032	0.378 ± 0.058	12.677	<0.001 *	0.061	<0.001 *	0.004 *
10	SLF.R	0.442 ± 0.026	0.422 ± 0.032	0.383 ± 0.067	11.98	<0.001 *	0.096	<0.001 *	0.003 *

* indicates statistical significance (*p* < 0.05, Bonferroni-corrected). FA, fractional anisotropy; HC, healthy controls; WML, white matter lesions; WML-VCIND, WML and non-dementia vascular cognitive impairment; WML-VaD, WML and vascular dementia.

**Table 3:** ROI level analysis MD results of WM skeleton partition.

WM Tracts		MD (Mean ± Std, ×10^−4^ mm^2^/s)			ANCOVA		Post Hoc Test (Bonferroni)		
							WML-VCIND/HC	WML-VAD/HC	WML-VAD/WML-VCIND
		HC (*n* = 37)	WML-VCIND (*n* = 33)	WML-VAD (*n* = 17)	F	*p*	*p*	*p*	*p*
1	ATR.L	7.446 ± 0.376	8.029 ± 0.501	9.329 ± 2.153	18.479	<0.001 *	0.057	<0.001 *	<0.001 *
2	ATR.R	7.706 ± 0.388	8.279 ± 0.563	9.593 ± 2.137	17.417	<0.001 *	0.067	<0.001 *	<0.001 *
3	FMA	7.953 ± 0.315	8.225 ± 0.355	8.906 ± 0.804	18.206	<0.001 *	0.049 *	<0.001 *	<0.001 *
4	FMI	7.645 ± 0.262	8.013 ± 0.444	8.840 ± 1.267	18.063	<0.001 *	0.055	<0.001 *	<0.001 *
5	IFOF.L	7.891 ± 0.33	8.440 ± 0.429	9.041 ± 1.054	19.713	<0.001 *	<0.001 *	<0.001 *	0.002 *
6	IFOF.R	7.995 ± 0.335	8.428 ± 0.374	9.112 ± 1.070	18.381	<0.001 *	0.006 *	<0.001 *	<0.001 *
7	ILF.L	7.867 ± 0.311	8.232 ± 0.427	8.568 ± 0.693	11.37	<0.001 *	0.003 *	<0.001 *	0.043 *
8	ILF.R	7.933 ± 0.332	8.268 ± 0.434	8.658 ± 0.738	9.533	<0.001 *	0.012 *	<0.001 *	0.021 *
9	SLF.L	7.324 ± 0.349	7.920 ± 0.448	8.337 ± 1.093	17.883	<0.001 *	<0.001 *	<0.001 *	0.065
10	SLF.R	7.395 ± 0.421	7.973 ± 0.440	8.565 ± 1.299	16.276	<0.001 *	0.002 *	<0.001 *	0.015 *

* indicates statistical significance (*p* < 0.05, Bonferroni-corrected). MD, mean diffusivity; HC, healthy controls; WML, white matter lesions; WML-VCIND, WML and non-dementia vascular cognitive impairment; WML-VaD, WML and vascular dementia.

**Table 4:** ROI level analysis AD results of WM skeleton partition.

WM Tracts		AD (Mean ± Std, ×10^−3^ mm^2^/s)			ANCOVA		Post Hoc Test (Bonferroni)		
							WML-VCIND/HC	WML-VAD/HC	WML-VAD/WML-VCIND
		HC (*n* = 37)	WML-VCIND (*n* = 33)	WML-VAD (*n* = 17)	F	*p*	*p*	*p*	*p*
1	ATR.L	1.112 ± 0.039	1.175 ± 0.052	1.301 ± 0.214	18.364	<0.001 *	0.036 *	<0.001 *	<0.001 *
2	ATR.R	1.144 ± 0.041	1.205 ± 0.063	1.332 ± 0.218	15.652	<0.001 *	0.059	<0.001 *	<0.001 *
3	FMA	1.477 ± 0.044	1.481 ± 0.045	1.519 ± 0.057	1.708	0.188			
4	FMI	1.226 ± 0.036	1.254 ± 0.044	1.299 ± 0.081	12.632	<0.001 *	0.071	<0.001 *	0.010 *
5	IFOF.L	1.219 ± 0.037	1.275 ± 0.043	1.300 ± 0.085	14.695	<0.001 *	<0.001 *	<0.001 *	0.284
6	IFOF.R	1.234 ± 0.041	1.268 ± 0.039	1.313 ± 0.074	13.16	<0.001 *	0.013 *	<0.001 *	0.009 *
7	ILF.L	1.200 ± 0.043	1.230 ± 0.049	1.245 ± 0.057	4.291	0.017			
8	ILF.R	1.208 ± 0.040	1.237 ± 0.051	1.258 ± 0.061	4.165	0.019			
9	SLF.L	1.084 ± 0.044	1.145 ± 0.052	1.168 ± 0.098	12.393	<0.001 *	<0.001 *	<0.001 *	0.603
10	SLF.R	1.108 ± 0.051	1.166 ± 0.049	1.206 ± 0.108	12.505	<0.001 *	0.001 *	<0.001 *	0.119

* indicates statistical significance (*p* < 0.05, Bonferroni-corrected). AD, axial diffusivity; HC, healthy controls; WML, white matter lesions; WML-VCIND, WML and non-dementia vascular cognitive impairment; WML-VaD, WML and vascular dementia.

**Table 5:** ROI level analysis RD results of WM skeleton partition.

WM Tracts		RD (Mean ± Std, ×10^−4^ mm^2^/s)			ANCOVA		Post Hoc Test (Bonferroni)		
							WML-VCIND/HC	WML-VAD/HC	WML-VAD/WML-VCIND
		HC (*n* = 37)	WML-VCIND (*n* = 33)	WML-VAD (*n* = 17)	F	*p*	*p*	*p*	*p*
1	ATR.L	5.607 ± 0.396	6.171 ± 0.527	7.490 ± 2.169	18.064	<0.001 *	0.076	<0.001 *	<0.001 *
2	ATR.R	5.839 ± 0.403	6.396 ± 0.553	7.729 ± 2.125	17.883	<0.001 *	0.077	<0.001 *	<0.001 *
3	FMA	4.545 ± 0.335	4.931 ± 0.431	5.762 ± 1.145	18.384	<0.001 *	0.029 *	<0.001 *	<0.001 *
4	FMI	5.335 ± 0.270	5.748 ± 0.500	6.762 ± 1.504	18.392	<0.001 *	0.069	<0.001 *	<0.001 *
5	IFOF.L	5.744 ± 0.383	6.288 ± 0.498	7.056 ± 1.188	19.17	<0.001 *	0.002 *	<0.001 *	0.001 *
6	IFOF.R	5.822 ± 0.350	6.300 ± 0.454	7.103 ± 1.255	17.586	<0.001 *	0.009 *	<0.001 *	<0.001 *
7	ILF.L	5.802 ± 0.301	6.198 ± 0.439	6.626 ± 0.797	13.693	<0.001 *	0.003 *	<0.001 *	0.012 *
8	ILF.R	5.861 ± 0.343	6.215 ± 0.468	6.700 ± 0.836	10.817	<0.001 *	0.016 *	<0.001 *	0.007 *
9	SLF.L	5.565 ± 0.360	6.156 ± 0.478	6.668 ± 1.192	18.159	<0.001 *	0.001 *	<0.001 *	0.028 *
10	SLF.R	5.552 ± 0.431	6.131 ± 0.486	6.816 ± 1.438	16.102	<0.001 *	0.005 *	<0.001 *	0.009 *

* indicates statistical significance (*p* < 0.05, Bonferroni-corrected). RD, radial diffusivity; HC, healthy controls; WML, white matter lesions; WML-VCIND, WML and non-dementia vascular cognitive impairment; WML-VaD, WML and vascular dementia.

**Table 6:** Correlation analysis results between the DTI index in ROI and MoCA (*r* value).

WM Tracts		Correlation between FA of WM Tracts and MoCA (*r* Value)	Correlation between MD of WM Tracts and MoCA (*r* Value)	Correlation between AD of WM Tracts and MoCA (*r* Value)	Correlation between RD of WM Tracts and MoCA (*r* Value)
1	ATR.L	0.536 *	−0.571 *	−0.562 *	−0.570 *
2	ATR.R	0.529 *	−0.544 *	−0.527 *	−0.546 *
3	FMA	0.618 *	−0.584 *	−0.117	−0.620 *
4	FMI	0.570 *	−0.584 *	−0.489 *	−0.589 *
5	IFOF.L	0.567 *	−0.645 *	−0.546 *	−0.635 *
6	IFOF.R	0.547 *	−0.569 *	−0.444 *	−0.580 *
7	ILF.L	0.617 *	−0.595 *	−0.360 *	−0.644 *
8	ILF.R	0.527 *	−0.501 *	−0.294	−0.540 *
9	SLF.L	0.509 *	−0.651 *	−0.583 *	−0.642 *
10	SLF.R	0.505 *	−0.600 *	−0.542 *	−0.594 *

* indicates that the DTI index of the corresponding ROI is significantly correlated with MoCA. MoCA, the Montreal Cognitive Assessment.

## Data Availability

The datasets generated and analyzed during the present study are available from the corresponding author on reasonable request.
